# The Moderating Role of Psychological Safety in the Relationship between Job Embeddedness, Organizational Commitment, and Retention Intention among Home Care Attendants in Taiwan

**DOI:** 10.3390/healthcare11182567

**Published:** 2023-09-17

**Authors:** Min-Yen Chang, Chih-Kuang Fu, Chi-Fu Huang, Han-Shen Chen

**Affiliations:** 1 Department of Accounting, Jiaxing University, Jiaxing 314001, China; mingyen0223@zjxu.edu.cn; 2 Department of International Health Industry Management, Chung Shan Medical University, Taichung City 40201, Taiwan; joshfu8189@gmail.com (C.-K.F.); geek0857@gmail.com (C.-F.H.); 3 Department of Health Industry Technology Management, Chung Shan Medical University, Taichung City 40201, Taiwan; 4 Department of Medical Management, Chung Shan Medical University Hospital, No. 110, Section 1, Jianguo North Road, Taichung City 40201, Taiwan

**Keywords:** home care services, job embeddedness, organizational commitment, retention intention, psychological safety

## Abstract

As Taiwan’s population ages, the need for long-term care, such as home care, is increasing due to improved medical services and longer life expectancy; however, the current coverage rate for home care services is only 50%, highlighting the importance of retaining home care workers. This study applies job embeddedness, organizational commitment, and psychological safety as variables to explore the retention intention of Taiwan’s home care workers. A questionnaire survey was distributed among home care workers using convenience sampling, resulting in 547 collected questionnaires, of which 458 were valid. Data analysis was conducted with SPSS 22.0 and AMOS 22.0, and a structural equation model (SEM) was used to test the hypotheses. Our findings suggest that job embeddedness has a positive impact on organizational commitment (γ = 0.649, γ = 0.607, and γ = 0.628; *p* < 0.001) and retention intention (γ = 0.253, γ = 0.242, γ = 0.271; *p* < 0.001), similar to organizational commitment (γ = 0.721, *p* < 0.001). Additionally, organizational commitment mediates job embeddedness and retention intention, while psychological safety moderated organizational commitment and retention intention (β = 0.639; *p* < 0.001). This study aims to provide information for the development of more effective human resource policies and contribute to practical advancements in the home care service environment and management structure of home care organizations. By analyzing and exploring the main factors contributing to home care workers’ retention intention, we hope to enhance the overall benefits of home service organizations and the industry.

## 1. Introduction

In 2018, Taiwan’s elderly population comprised over 14% of the total population, officially marking the country’s entry into an aged society [1]. Projections indicate that by 2025, this proportion will surpass 20%, indicating the transition to a super-aged society [1]. The aging index, which measures the ratio of individuals aged 65 and above to those below 14, has risen from 43.01% in May 2002 to 139.71% in May 2022 [2]. Approximately 30% of elderly individuals in Taiwan live alone or reside solely with their spouses [3].

The Ministry of Health and Welfare [4] reveal that individuals require an average of around 9 years of care during old age, considering the gap between the average life expectancy (81.3 years) and healthy life expectancy (72.4 years) in 2020. (Healthy life expectancy refers to the average number of years a person is expected to live in good health without significant restrictions or disabilities affecting their daily functioning and quality of life. It measures the overall health and well-being of a population by considering both the length of life and the quality of life during those years.) Currently, the coverage rate of home care services in Taiwan exceeds 56%, benefiting from over 480,000 individuals receiving long-term care services; however, the demand for such care surpasses 850,000 individuals, highlighting the pressing need for increased investment in home care human resources [3].

The research report released in 2020 by the National Development Council highlights the projected accelerated pace of population aging in Taiwan compared to countries such as Japan, Germany, and the United States. According to the report, Taiwan’s total dependency ratio was recorded at 40.1% in 2020, with the elderly dependency ratio standing at 22.5%. This indicates that approximately five working-age adults are responsible for supporting one elderly individual. The projections for 2070 estimate a significant increase in the total dependency ratio to 102.0%, with the elderly dependency ratio reaching 84.0%. As a result, it is expected that an average of 1.2 working-age adults will need to assume the caregiving responsibility for each elderly person. These statistics underscore the escalating burden of care due to the continuous rise in the elderly dependency ratio, emphasizing the critical significance of this pressing concern in Taiwan.

Nevertheless, the challenge of population aging extends beyond Taiwan and represents a global phenomenon that has reached an irreversible stage. Developed Western nations, in particular, recognized the need for long-term care services in the post-World War II era. It became evident that relying solely on medical security funds for funding long-term care was inadequate. This inadequacy arose from the requirement of disabled older individuals for more comprehensive life and health care beyond medical interventions. Consequently, the landscape of long-term care shifted towards non-institutionalized home-based and community-based services, which have consistently expanded their availability. Japan, located in close proximity to Taiwan, transitioned into a super-aged society in 2006. However, the scarcity of resources and policy constraints have led to significant social challenges in terms of home care. Katsuro Iguchi’s [5] research shed light on the exponential increase in “family care mental illness” and “family care homicides” reaching an alarming scale. This phenomenon has resulted in heightened fatigue among Japan’s young population, working-age individuals, elderly citizens, and care recipients. The far-reaching consequences of limitations in family care highlight the impact of population aging on Japanese society, even in highly industrialized nations, posing severe and urgent issues.

According to Control Yuan’s [6] assessment, Taiwan is currently confronting a persistent shortage of long-term care workers. In 2020, the active involvement of certified caregivers in long-term care services was merely 25%, which decreased to 21% by 2017. The implementation of the “Ten-Year Long-Term Care Plan 2.0” in 2018 exacerbated the shortage, resulting in a deficit of over 8000 workers by 2020. Despite collaborative efforts between the Ministry of Health and Welfare and the Ministry of Labor to establish vocational training programs for caregiving professions in recent years, the number of care workers has rapidly escalated from 53,212 individuals at the end of 2019 to over 87,000 by the end of 2021 [4]. However, the caregiving workforce continues to struggle to keep up with the growing demands of eldercare, underscoring the urgent need for an increased number of care workers in the future.

In light of the surge in demand for long-term care services in Taiwan, despite the government’s endeavors to establish numerous community-based service facilities and allocate resources, an inherent supply–demand imbalance persists in meeting the needs of the burgeoning population requiring long-term care. Upon closer examination, it becomes evident that there has been a lack of proportional growth in the number of caregivers. Consequently, individual caregivers face an increasingly demanding burden in terms of average service hours and caseload compared to previous periods.

Home care workers provide a range of caregiving services either in clients’ homes or non-residential care facilities. Their duties encompass accompanying clients to appointments, meal preparation, bathing, assistance with eating, support during outings and shopping, providing personal hygiene assistance, and offering companionship. These workers undergo training to effectively address the diverse needs of their clients and must possess adaptability to various situations and environments. However, they also encounter workplace violence, psychological distress, heavy workloads, stress, fatigue, and pressure [7]. These factors contribute to their propensity to leave their positions [8], and physical or mental injuries can lead to involuntary resignations. The importance of job significance and working conditions are strong indicators of their intention to stay [9].

Gao et al. [10] conducted research that demonstrates a notable correlation between retention, salary, and job pressure in the realm of home care service workers. The study reveals that local workers’ identification with their jobs positively influences their inclination to remain employed, while foreign workers are more influenced by organizational support as a deciding factor. This support encompasses aspects such as organizational culture, language assistance, a sense of belonging, and appropriate workloads. Furthermore, Costello et al. [11] conducted a systematic review and analysis to examine the occurrence and association of stress and burnout among staff working in long-term care facilities for individuals with dementia. In addition, Xiao et al. [12] proposed that the retention of elderly caregivers is influenced by a combination of personal, organizational, and social factors. Notably, the shortage of elderly caregivers has been exacerbated during the COVID-19 outbreak, underscoring the need for elderly care institutions to create a supportive work environment that enhances staff retention and attracts skilled talent.

Former research mainly focuses on job satisfaction, commitment, and choices to predict turnover [13], which has been proven to be unreliable [14]. There is a discrepancy between job satisfaction and turnover rates, with a 12% gap between turnover intentions and actual rates [15]. Mitchell et al. [16] proposed the concept of “job embeddedness” to explain turnover by emphasizing connections and associated costs. Higher levels of job embeddedness decrease the likelihood of turnover. In comparison to commitment and satisfaction, job embeddedness offers a more comprehensive analysis of turnover [17]. 

Additionally, numerous companies value employees’ perceptions and values. For example, Altmann [18] linked frontline employees’ perception of the work environment to psychological safety. When organizations establish a positive environment that allows employees to reach their full potential, psychological safety becomes essential [19]. Nurturing positive psychological safety fosters employee identification, enhances job satisfaction, promotes a willingness to contribute, and improves retention intention.

Previous research on the turnover or retention of home care workers has predominantly focused on examining the relationship between various variables and turnover or retention. However, there is a need to enhance the description of how different aspects interact with each other since these factors are not completely independent or unrelated. Therefore, this study makes a significant contribution by examining organizational factors that facilitate job embedding and analyzing their impact on the retention intention of home care workers. Additionally, it investigates the influence of psychological safety on their intention to stay. The findings provide valuable insights into retention-related aspects and offer guidance for organizations seeking to improve the work environment for home care workers.

## 2. Materials and Methods

### 2.1. Research Framework

**Figure 1:**
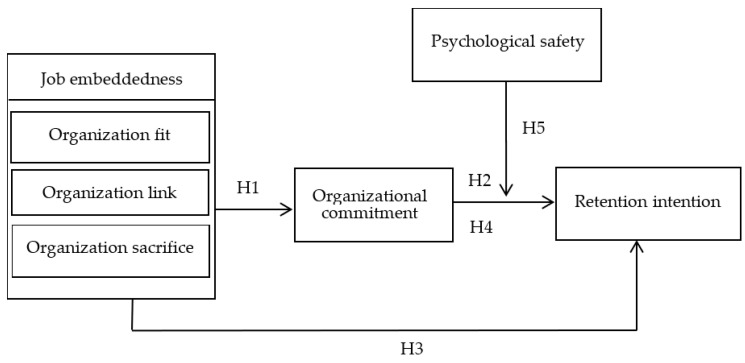
The conceptual framework and hypotheses.

Drawing upon the mentioned research findings, Figure 1 illustrates the conceptual framework, including job embeddedness (fit, linkage, sacrifice), organizational commitment, psychological safety, and retention intention.

### 2.2. Research Hypotheses 

#### 2.2.1. Job Embeddedness

The evaluation of job embeddedness involves assessing links, fit, and sacrifice. Links encompass interactions within and beyond the scope of work. Fit relates to the alignment with the organizational culture and community. Sacrifice entails the costs and missed prospects associated with leaving the organization [20]. Both organizational and community embeddedness have a notable impact on the organization [21]. This particular research focuses on home care workers, examining their organizational fit, link, and sacrifice without considering the broader context of embeddedness beyond work.

#### 2.2.2. Organizational Commitment

The commitment displayed by individuals reflects their dedication to the organization, indicating organizational commitment [22]. Strong commitment leads to increased effort and decreased turnover [23]. Recognizing employees’ contributions fosters a positive attitude and reduces the likelihood of attrition [24]. Meyer and Allen [25] classified organizational commitment into three types: affective commitment, continuance commitment, and normative commitment. Affective commitment refers to emotional attachment and loyalty to the organization arising from a strong sense of belonging and identification. Continuance commitment is driven by economic or practical reasons, even without a strong emotional bond to the organization. Normative commitment stems from moral obligations and a sense of responsibility. Employees with normative commitment choose to stay because they consider fulfilling their commitment to the organization a moral duty.

Previous studies have emphasized the impact of affective commitment on job performance [26,27,28]. Committed employees tend to remain with the organization when their personal and organizational values align [29]; building strong employee relationships strengthens affective commitment [23]. Normative commitment involves loyalty, responsibility, and reciprocation of organizational investments, reflecting individuals’ sense of obligation and loyalty [26,30]. Continuance commitment is based on employees considering the costs, risks, and financial implications of leaving and weighing the decision [23]. Some individuals exhibit continuance commitment due to external factors, such as compensation and retirement benefits, without a strong alignment with organizational values and goals. Organizational commitment plays a crucial role in job performance and employee engagement [31], with consistent studies showing a robust connection between organizational commitment and job performance [32].

Previous research has highlighted a positive correlation between job embeddedness and organizational commitment [33,34,35]. Organizational commitment acts as a mediator between job embeddedness and turnover intentions [35]. By building on the literature mentioned above, this study proposes the following hypotheses:

**Hypothesis 1-1 (H1-1).**  *Higher levels of organizational fit among home care workers correspond to greater organizational commitment.*

**Hypothesis 1-2 (H1-2).**  *Increased organizational linkage among home care workers corresponds to higher organizational commitment.*

**Hypothesis 1-3 (H1-3).**  *Heightened organizational sacrifice among home care workers corresponds to higher organizational commitment.*

#### 2.2.3. Retention Intention

Johari et al. [36] defined the stay intention as employees’ desire to continue their employment, while Coetzee and Stoltz [37] suggested that the stay intention represents employees’ willingness to remain and collaborate within the organization. The stay intention, as noted by Al-Hamdan et al. [38], reflects an attitude toward sustainable work. It is influenced by factors such as burnout, commitment, involvement, satisfaction, compensation, and development opportunities [39,40]. Job satisfaction has a strong connection to staying intention, a crucial predictor of employee retention, as found by Mardanov [41]. Rodríguez-Fernández et al. [42] demonstrated the negative impact of burnout on well-being and staying intention [13]. Similarly, organizational commitment strongly correlates with staying intention [43].

Employees who demonstrate a willingness to stay exhibit commitment and satisfaction, facilitated by supportive work environments, suitable schedules, and positive interactions. Peer support mitigates criticism [44], while positive social interactions contribute to higher retention rates [45]. Perryer et al. [46] established a link between organizational commitment and retention intention, indicating that higher organizational commitment enhances retention intention. Positive factors such as selection, evaluation, promotions, training, career prospects, communication, trust, and fairness contribute to increased commitment and retention [47]. In their survey of Irish nursing staff in the National Health Service, Bell and Sheridan [43] examined job stress, satisfaction, burnout, and commitment. They identified organizational commitment as the primary predictor of intention to remain in nursing work. Based on the existing literature, the following hypothesis is proposed:

**Hypothesis 2 (H2).**  *The higher the home care workers’ commitment to the organization, the greater their retention intention.*

Job embeddedness is vital in influencing employee retention and turnover [48]. It also acts as a significant mediator of employees’ intention to stay [49]. Coetzer et al. [50] found a negative correlation between job embeddedness and turnover intention, while Larkin et al. [51] stressed the importance of reinforcing job embeddedness through retention policies to facilitate proactive employee integration. Previous studies consistently support the link between job embeddedness and employee retention [16,52], indicating that higher job embeddedness reduces turnover likelihood and enhances employee retention. Zhang et al. [14] discovered that job embeddedness predicts employee performance. Moreover, research suggests that both job satisfaction and organizational commitment influence individuals’ intention to stay with the organization [53]. Meaningful work, teamwork, and positive relationships foster commitment and enhance retention intention [16]. Dissatisfaction due to inadequate alignment between the organization and its employees undermines retention intention [54]. Traditional attitude models address concerns about turnover and economic loss by emphasizing the relationship between economic benefits, job satisfaction, and retention intention [8]. Additionally, other organizational factors such as job stability, promotions, and relationships affect employees’ decisions. Given the cited literature, we propose the following hypotheses:

**Hypothesis 3-1 (H3-1).**  *The higher the alignment between home care workers and the organization, the higher their retention intention.*

**Hypothesis 3-2 (H3-2).**  *The greater the organizational connectivity of home care workers, the higher their retention intention.*

**Hypothesis 3-3 (H3-3).**  *The higher the organizational sacrifice of home care workers, the greater their retention intention.*

#### 2.2.4. Psychological Safety

Psychological safety denotes trust among employees in the workplace [55]. Edmondson [56] stressed the significance of fostering a psychologically secure work environment that encourages open expression, feedback, collaboration, risk-taking, and experimentation to facilitate learning. Nahrgang et al. [57] defined psychological safety as a supportive environment characterized by commitment, empowerment, involvement, and communication. Leonard-Barton and Swap [58] suggested that psychological safety impacts learning outcomes and job performance. Various studies have established a link between psychological safety and accelerated learning and innovation [59,60]. Prioritizing leadership programs that foster inclusivity, empathy, and supportive guidance from leaders can cultivate psychological safety [61].

Committed individuals exhibit proactive behaviors toward the organization, such as going above and beyond, making sacrifices, demonstrating loyalty, and expressing a desire to continue [62]. Managing commitment, satisfaction, and individual factors such as stress and burnout are vital for talent retention [63]. Robinson et al. [64] discovered that organizational sacrifice and community connections impact commitment and turnover. Organizational sacrifice negatively affects turnover, while community connections have a positive effect. Based on these scholars’ findings, we propose the following hypotheses:

**Hypothesis 4-1 (H4-1).**  *Enhanced organizational fit boosts retention intention *via* organizational commitment.*

**Hypothesis 4-2 (H4-2).**  *Enhanced organizational linkage boosts retention intention *via* organizational commitment.*

**Hypothesis 4-3 (H4-3).**  *Enhanced organizational sacrifice boosts retention intention *via* organizational commitment.*

Low psychological safety causes absenteeism, negative attitudes, and employee conflicts [65]. In contrast, higher psychological safety encourages employee engagement, idea sharing, and collaboration, leading to better organizational performance [56]. Research shows a link between psychological safety, job resources, and affective commitment [66], where affective commitment significantly influences turnover intentions. Psychological safety contributes to a positive work environment [67] and promotes innovation and job effectiveness [68].

Gao et al. [69] discovered that superior–subordinate relationships impact employee commitment, with dyadic exchange playing a crucial role; psychological safety moderates this connection and is associated with dedication and the intention to stay. Thus, we suggest the following hypothesis:

**Hypothesis 5 (H5).**  *Psychological safety enhances the link between organizational commitment and retention intention.*

### 2.3. Research Questionnaire Design

This study used a self-administered survey. We conducted an expert review to guarantee the clarity and accuracy of the questions. Nine experts, each with more than 10 years of experience in academia and home care services, actively participated and provided valuable feedback, which significantly enhanced the questionnaire. We conducted a pilot test with 70 questionnaires, obtaining 61 valid responses. Item analysis and reliability assessment were conducted for each dimension, and this rigorous process allowed us to finalize the questionnaire design.

The questionnaire comprises five sections. Section 1 comprises 20 items and assesses job embeddedness, incorporating scales from Mitchel et al. [16], Lee et al. [70], and Holtom and Inderrieden [71] to measure organizational fit, organizational links, and organizational sacrifice. Section 2 has 11 items, centering on organizational commitment using Meyer and Allen’s [25] dimensions of affective, continuance, and normative commitment. Section 3 comprises five items that evaluate the retention intention and adopts the intention to stay scale developed by Coetzee and Stoltz [37]. Section 4 uses six items to address psychological safety and utilizes the Psychological Safety Scale by Edmondson [56]. Section 5 collects demographic information, including gender, education level, years of experience in caregiving, previous types of caregiving work, longest period of caregiving work, salary, and age. All items, excluding demographic variables, are assessed using a 7-point Likert scale, with responses ranging from “strongly disagree” (1) to “strongly agree” (7).

### 2.4. Sample and Data Collection

In order to ensure accuracy, convenience, comprehensive coverage, and compliance with COVID-19 pandemic guidelines, as well as adherence to epidemic prevention policies and public sentiment, this study employed a diverse range of strategies for distributing and collecting the questionnaires. A combination of physical and electronic questionnaires was utilized, initially approaching local home care service organizations in Taiwan for assistance in either distributing physical paper questionnaires or disseminating electronic online questionnaires to their affiliated staff, based on the feasibility of each method at the time. Concurrently, the research team proactively distributed survey questionnaires through prominent online communities for nationwide home care service providers, including popular platforms such as Facebook and LINE.

Although platforms such as Facebook and LINE are useful for gathering data, it is important to recognize that interactions in online communities can potentially influence respondents and result in biased or conforming responses. Internet surveys may be affected by various factors that introduce bias and impact the study’s validity, such as non-representative samples [72], lack of researcher probing, reliance on internet users only, and the potential for duplicate responses [73]. Additionally, concerns about privacy and the visibility of online interactions significantly affect individuals’ willingness to disclose information or respond to sensitive inquiries. Therefore, researchers must acknowledge and address these influential factors to ensure the acquisition of valid and representative data. Our aim was to adopt this approach to track respondent eligibility and representativeness, ultimately increasing the number of effectively collected questionnaires during the challenging epidemic period.

According to the purpose of this study and hypothesis test, the data were statistically analyzed using structural equation modeling. Wu suggested that the optimal sample size of the structural equation should be determined by the number of questions, and the optimal sample size-to-questions ratio should be between 10:1 and 15:1 [74]. Since the questionnaire comprised 42 items, the ideal sample size was 420–630. The data were collected in March and April 2022. A total of 547 responses were collected. After excluding 76 invalid questionnaire responses, a total of 458 valid questionnaire responses remained for the analysis.

Regarding gender, the respondents included 143 males (31.22%) and 315 females (68.88%). The education levels comprised 215 participants with high school or below (46.72%) and 157 with vocational college (34.28%) education. The age groups included 166 participants aged 50–59 (36.25%), 118 aged 40–49 (25.76%), 77 aged 30–39 (16.81%), 61 aged 60 and above (13.32%), and 36 respondents aged 20–29 (7.86%). Regarding caregiving experience, 165 participants had 1–3 years (36.03%) of experience, while 85 had less than one (18.56%). The respondents’ average monthly income ranged between NTD 30,001 and 40,000 (148 participants, or 36.03%) and NTD 40,001 and 50,000 (121 participants, or 26.42%). The most prevalent caregiving work type included home care service agencies, with 283 participants (61.78%), and one-on-one nursing in hospital wards, with 62 participants (13.54%). The demographic analysis of the sample is presented in Table 1.

### 2.5. Techniques for Analyzing the Data

This study employed structural equation modeling (SEM) to assess the causal connections between latent variables. SEM was utilized for analysis, while statistical software packages, such as SPSS Statistics 22.0 and IBM SPSS Amos 22.0 (IBM Corporation, Armonk, NY, USA), served as valuable tools for testing and refining theoretical models, enabling the examination of causal relationships among variables based on statistical dependencies. Due to the influence of latent variables, which cannot be directly measured, on consumer behavior, the indirect measurement of observable variables becomes necessary. SEM offers a means to explore and elucidate these relationships.

SEM is considered a large sample technique that typically requires a minimum sample size of 200. The sample size is typically determined by three factors: the type of distribution of observed variables, the complexity of the model, and the estimation method employed [75]. Numerous researchers have applied SEM to investigate various research topics [76,77,78]. Accordingly, this study opted for SEM to investigate the relationship between job embeddedness, organizational commitment, psychological safety, and retention intention among home care attendants in Taiwan.

## 3. Results

### 3.1. Evaluation of the Measurement Model: Test Results

This study employed a two-stage analysis with (1) confirmatory factor analysis (CFA) to measure scales and (2) structural model analysis. After verifying data normality, CFA was conducted to examine the relationships between observed variables and latent factors [79]. CFA is generally used to assess the validity and reliability of unobserved latent factors [79,80]. Since this study used questionnaires developed by other researchers, CFA was employed to assess whether our measurement was suitable for the target population. Following Hair et al. [79], this study evaluated Cronbach’s alpha, factor loadings, composite reliability (CR), average variance extracted (AVE), and discriminant validity.

Table 2 presents the analysis results of the reliability and validity of the dimensions. Our findings show that item loadings in each construct ranged from 0.732 to 0.928, meeting the established criterion of exceeding 0.5 in previous research [79]. Cronbach’s alpha values for each construct were as follows: job embeddedness (0.912), organizational commitment (0.894), intention to stay (0.896), and psychological safety (0.864). These values surpassed the recommended threshold of 0.7 suggested by Hair et al. [79], indicating strong internal consistency in the research model. AVE values for each construct ranged from 0.692 to 0.828, surpassing the suggested value of 0.5 [79]. These results indicate robust convergent validity for each construct. Based on the above explanations, this study includes a table demonstrating that the constructs of the questionnaire meet convergent validity and CR requirements, thus showcasing excellent internal quality.

Table 3 displays the correlation coefficients and the square root of the AVE for the measurement model. To assess discriminant validity, it is recommended to adhere to the guidelines established by Fornell and Larcker [81], where the square root of the AVE should exceed the correlation coefficient between variables to ensure satisfactory discriminant validity. As shown in Table 3, all correlation coefficients between variables were found to be lower than the square root of the AVE, thereby fulfilling the criteria proposed by Fornell and Larcker [81]. Thus, this demonstrates that the variables examined in this study exhibit adequate discriminant validity.

### 3.2. Structural Model Evaluation

This study’s structural model analysis yields the following findings: χ2/df = 3.891, standardized root mean square residual (SRMR) = 0.069, root mean square error of approximation (RMSEA) = 0.063, goodness of fit index (GFI) = 0.903, normed-fit-index (NFI) = 0.912, incremental fit index (IFI) = 0.908, and comparative fit index (CFI) = 0.913. These findings demonstrate the goodness-of-fit indices for the research model. ((1) SRMR (standardized root mean square residual) is an index that measures the dissimilarity between the observed covariance matrix and the model’s predicted covariance matrix. It provides insights into the average level of residual variance and covariances within the model. (2) RMSEA (root mean square error of approximation) quantifies the average discrepancy between the observed and model-predicted covariance matrices per degree of freedom. This estimate reflects the goodness of fit of the model to the observed data, where lower values indicate a superior fit. (3) GFI (goodness of fit index) evaluates the model’s ability to explain the variance and covariance in relation to the overall observed variance and covariance. The GFI indicates the overall fit of the model to the data, with values closer to 1 indicating a strong fit. (4) NFI (normed fit index) compares the discrepancy between the model-predicted and null model covariance matrices to the discrepancy between the observed and null model covariance matrices. Its values range from 0 to 1, where higher values suggest a better fit. (5) IFI (incremental fit index) compares the improvement in fit between the proposed model and the null model against the expected improvement if the model fits perfectly. The IFI ranges from 0 to 1, with higher values indicating a more favorable fit. (6) CFI (comparative fit index) compares the fit of the hypothesized model with a baseline model, typically the null model. The CFI ranges from 0 to 1, with values closer to 1 indicating a better fit. A CFI value of 0.90 or higher is generally considered indicative of an acceptable fit.) Table 4 presents the path coefficients and hypothesis verification for the research model.

Based on the data in Table 4, the path coefficients between organizational fit and organizational link, organizational sacrifice, and organizational commitment are all significantly positive (γ = 0.649, γ = 0.607, and γ = 0.628; *p* < 0.001). These results indicate that increased levels of organizational fit, organizational link, and organizational sacrifice among home care workers are associated with higher organizational commitment; therefore, the findings support Hypotheses H1–H3. The path coefficient between organizational commitment and retention intention is significantly positive (γ = 0.721, *p* < 0.001), suggesting that higher levels of organizational commitment among home care workers are linked to an increased retention intention; hence, Hypothesis 2 is supported. Furthermore, the path coefficients between organizational fit, organizational link, organizational sacrifice, and retention intention are significant (γ = 0.253, γ = 0.242, γ = 0.271; *p* < 0.001). These findings indicate a positive relationship between higher levels of organizational fit, organizational link, organizational sacrifice, and retention intention among home care workers. Therefore, Hypotheses H3-1 to H3-3 are supported.

### 3.3. Mediation Effects Testing

Table 5 indicates that organizational fit exhibits a direct effect of 0.258 on retention intention, which is smaller than the indirect effect of 0.469. This result suggests a mediation effect, thereby supporting Hypothesis H4-1. Similarly, organizational link demonstrates a direct effect of 0.249 on retention intention, which is smaller than the indirect effect of 0.442. This result indicates a mediation effect, supporting Hypothesis H4-2. Likewise, organizational sacrifice displays a direct effect of 0.274 on retention intention, which is smaller than the indirect effect of 0.455. This result suggests the presence of a mediation effect, supporting Hypothesis H4-3.

### 3.4. Moderation Effects Testing

This study analyzed how psychological safety affects the relationship between “organizational commitment and retention intention” using hierarchical regression analysis. The results are presented in Table 6. In regression model M3, “organizational commitment × psychological safety”, the explained variance (R^2^) increased to 0.268, signifying a 0.018 change in explained variance (ΔR^2^). The regression coefficient was significant (β = 0.639; *p* < 0.001), indicating a moderate effect of psychological safety on “organizational commitment and retention intention”. Furthermore, the sample was divided into two groups based on high and low levels of psychological safety (using the mean as the cutoff point).

**Figure 2:**
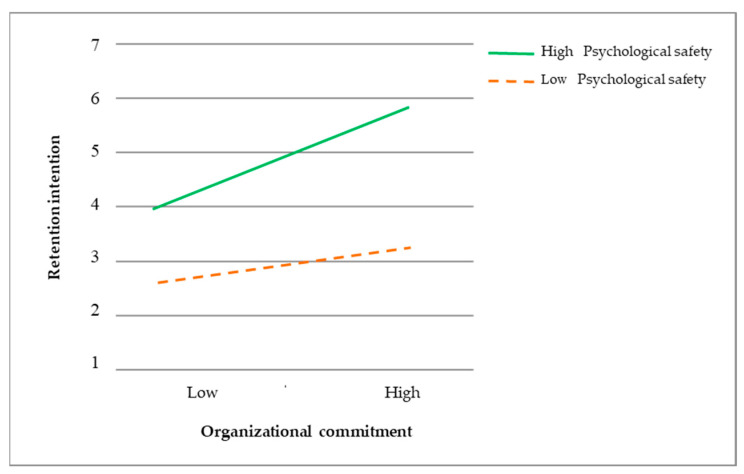
Organizational commitment, retention intention, and psychological safety interact.

Figure 2 exhibits the plotted regression model lines for organizational commitment and retention intention. It reveals that heightened psychological safety enhances the moderate impact of organizational commitment on retention intention among home care workers. Conversely, lower psychological safety amplifies the moderate effect of organizational commitment on retention intention. The line slopes indicate that improved psychological safety among home care workers correlates with a more robust effect on “organizational commitment and retention intention”.

## 4. Discussion

This study aimed to examine the associations between job embeddedness, organizational commitment, and retention intentions among home care workers. Data analysis revealed several key findings. 

Firstly, job embeddedness, including factors such as fit, linkage, and sacrifice, positively impacts organizational commitment. This aligns with previous research by Robinson et al. [64], emphasizing the relationship between high embeddedness and commitment. Conversely, feelings of undervaluation or misalignment decrease commitment. Supportive supervisors who value employees’ strengths enhance identification and commitment, while disharmony and exclusion have a negative effect. Encouraging participation fosters interaction and commitment. Unfair compensation and future concerns undermine commitment, but recognizing value, providing growth opportunities, and equitable benefits strengthen it.

Secondly, organizational commitment was found to enhance the intention to stay among home care workers, consistent with studies by De Gieter et al. [82] and Hansel et al. [83]. Boosting commitment fosters dedication to the organization. Emphasizing education, training, and building strong connections enhances the desire to stay.

Thirdly, strong job embeddedness increases retention intentions among home care workers, supporting previous research by Tanova and Holtom [52] and Robinson et al. [84]. Alignment between the organization and employees enhances the likelihood of staying. Additionally, organizational commitment partly mediates the relationship between job embeddedness and retention intention, as observed by Peachey et al. [85]. Cultivating a stronger organizational identification boosts the inclination to stay.

Lastly, psychological safety plays a significant moderating role for home care workers, as discovered by Kirk-Brown and Van Dijk [66]. A positive psychological safety climate enhances organizational commitment, retention intention, and support for institutional policies. Work–life balance, as emphasized by Deery and Jago [63], promotes satisfaction and commitment and reduces turnover. Prioritizing employee satisfaction is crucial for long-term retention. Karatepe and Vatankhah [86] highlighted attractive compensation, promotions, working conditions, skill development, and training for optimal performance. Job satisfaction drives continuous improvement.

In summary, the findings underscore the significance of job embeddedness, organizational commitment, and retention intentions among home care workers. The insights gained from the identified relationships and themes hold value for organizations aiming to promote commitment, address job embeddedness factors, and enhance retention. Cultivating a positive climate of psychological safety and prioritizing employee satisfaction is paramount for improving organizational commitment and performance. Specific recommendations can be outlined as follows: (1) Salary and benefits: home care organizations should contemplate the development of diverse schemes to provide win–win incentives tailored to the varying professional levels of home care services, enabling them to attract talent effectively. (2) Professional training arrangements: a proactive emphasis should be placed on the growth and training of caregivers. This approach not only enriches the talent pool within the organization but also reinforces caregiver job embeddedness, enhances organizational commitment, acknowledges caregiver psychological safety, increases willingness to remain in the organization, and subsequently fosters a competitive and virtuous cycle.

## 5. Conclusions

### 5.1. Management Implications

The research findings indicate that salary, benefits, security, and growth are vital for home care workers. Thorough planning is crucial for long-term success. First, agencies should offer tailored compensation and benefits to support caregiver growth and assignments, fostering mutual incentives. Second, caregiver growth and training should be prioritized to enhance human resource quality and case handling. This approach can improve brand reputation, competitiveness, and organizational commitment. Lastly, a positive psychological safety environment for trust and effective communication should be created among caregivers.

### 5.2. Limitations of the Study and Future Directions for Research

During the COVID-19 pandemic, the participation of home care workers in this study was affected by limitations in sample selection, potentially influencing the interpretation of the questionnaires due to the workers’ diverse backgrounds. Consequently, the study did not accurately capture the population proportions or the distribution of home care workers across different regions and administrative areas, leading to an incomplete understanding of the overall population correlations. The survey utilized a seven-point Likert scale and incorporated confidentiality measures. However, the constraints imposed by the pandemic and remote survey administration gave rise to concerns among participants regarding data handling, thereby introducing the possibility of measurement errors. Moreover, the notable limitation arises from the use of self-administered tools for data collection in this study. Given its potential to yield biased results, this method necessitates a thorough examination of its implications on the study’s findings.

## Figures and Tables

**Table 1:** Demographic analysis.

Variable	Description	Frequency (*n* = 458)	Percentage
Gender	Male	143	31.22%
	Female	315	68.88%
Age	20–29	36	7.86%
	30–39	77	16.81%
	40–49	118	25.76%
	50–59	166	36.25%
	60 or above	61	13.32%
Education Level	Junior high school or below	214	46.72%
	Junior college degree	157	34.28%
	Undergraduate degree	48	10.48%
	Master’s degree (inclusive) and above	39	8.52%
Personal Monthly Income (NTD)	Less than NTD 20,000	37	8.08%
	NTD 20,001–30,000	95	20.73%
	NTD 30,001–40,000	148	32.31%
	NTD 40,001–50,000	121	26.42%
	NTD 50,001–60,000	48	10.46%
	Above NTD 60,001	9	2.00%
Decades of Job Expertise	Less than 1 year	85	18.56%
	1~3 years	165	36.03%
	3~5 years	66	14.41%
	5~7 years	37	8.08%
	7~10 years	48	10.48%
	10 years or more (including)	57	12.44%
Types of Caregiving Services	Nursing home	19	4.15%
	Long-term care facility	36	7.86%
	Hospice	44	9.61%
	Hospital ward one-on-one care	62	13.54%
	Home care service agency	283	61.78%
	Other	14	3.06%

**Table 2:** Results of the factor loading, reliability, and validity.

Variables		Items	Standardized Factor Loadings	CR	AVE	Cronbach’s α
Job embeddedness	Organizational fit	1. My current job effectively utilizes my talents.	0.826 ***	0.915	0.782	0.912
		2. I believe I am well-suited for my current position in the home care organization.	0.848 ***			
		3. I perceive that the home care organization I work for highly values my employment.	0.825 ***			
		4. I appreciate the scheduling arrangement of my present job.	0.817 ***			
		5. I find satisfaction in collaborating with colleagues at the current home care organization.	0.829 ***			
		6. I derive fulfillment from my current role within the home care organization.	0.834 ***			
		7. My principles align with the philosophy upheld by this home care organization.	0.873 ***			
		8. This job draws upon my skills and abilities.	0.861 ***			
		9. I am a good fit for the corporate culture of this home care organization.	0.837 ***			
		10. In the future, I aspire to assume greater authority and responsibility within this home care organization.	0.881 ***			
	Organizational links	11. I frequently have positive interactions with my colleagues during work hours.	0.816 ***	0.846	0.714	
		12. Following work, I actively engage in conversations or activities alongside my colleagues.	0.772 ***			
		13. I discover that my colleagues actively participate together in the various events organized by our healthcare institution.	0.862 ***			
	Organizational sacrifice	14. My colleagues demonstrate a high level of respect towards me.	0.802 ***	0.892	0.725	
		15. The present salary is satisfactory to me.	0.815 ***			
		16. I hold the view that remaining employed at this home care establishment offers promising opportunities.	0.846 ***			
		17. Departing from this establishment would entail compromising the reliance others place on me.	0.828 ***			
		18. I am ready to enhance my job skills for the betterment of this home care institution.	0.803 ***			
		19. This job encompasses numerous benefits.	0.826 ***			
		20. The home care institution I am affiliated with offers commendable employee benefits.	0.871 ***			
Organizational commitment	Affective commitment	1. I plan to work at this home care facility for the entirety of my professional journey.	0.805 ***	0.862	0.692	0.894
		2. I am pleased to engage in conversations about the home care facility I am employed at with individuals external to the organization.	0.774 ***			
		3. I have confidence in my ability to seamlessly transition my loyalty to a different home care institution.	0.732 ***			
		4. This home care facility carries immense personal importance to me.	0.815 ***			
	Continuance commitment	5. Although I have not secured an alternate job yet, I fearlessly accept the repercussions of quitting my present role.	0.838 ***	0.927	0.801	
		6. Departing from this nursing home is an arduous task, making it challenging for me to submit my resignation, even if I desire to do so.	0.924 ***			
		7. Opting to resign from this nursing home would introduce numerous disruptions in my life.	0.891 ***			
		8. I will persist in working at this nursing home due to the substantial personal sacrifices associated with resigning and the inability of another organization to offer me all the current benefits I enjoy.	0.927 ***			
	Normative commitment	9. I will persist at this care facility as I am committed to upholding workplace ethics.	0.896 ***	0.874	0.793	
		10. Even with a more enticing job offer, I would not deem leaving this care institution as the correct choice.	0.863 ***			
		11. I have been instilled with the principle of remaining loyal to the company organization.	0.958 ***			
Retention intention		1. I am ready to stay at the present home care institution and take on the given assignments.	0.843 ***	0.865	0.706	0.896
		2. I firmly believe that serving in this institution is the correct decision.	0.827 ***			
		3. The existing work conditions and environment offered by this home care institution motivate me to continue with my tenure in the foreseeable future.	0.861 ***			
		4. Even if I were to terminate my involvement in the current case service, I would still not contemplate leaving this home care institution, and there would be a chance to transition to another institution.	0.842 ***			
		5. Pursuing my career in this home care institution would greatly aid my future career plans.	0.794 ***			
Psychological safety		1. My colleagues at the home care organization I work for are inclined to share information rather than hoard it.	0.820 ***	0.917	0.828	0.864
		2. Collaboration and teamwork take precedence among the team members at the home care organization I work for.	0.881 ***			
		3. The team members at the home care organization I work for exert influence on one another.	0.915 ***			
		4. Communication and discussion regarding work-related matters occur among the team members at the home care organization I work for.	0.849 ***			
		5. The team members at the home care organization I work for are readily understood and accepted by their peers.	0.853 ***			
		6. Even when expressing immature perspectives, the team members at the home care organization I work for receive due consideration.	0.928 ***			

Note: CR: composite reliability; AVE: average variance extracted. *** *p* < 0.001.

**Table 3:** Correlation coefficients and the square root of AVE for the measurement model.

	Mean	Standard Deviation	1	2	3	4	5	6
Organizational fit	5.102	0.719	**0.725**					
Organizational links	5.712	1.118	0.232 **	**0.799**				
Organizational sacrifice	5.003	1.002	0.717 **	0.237 **	**0.810**			
Organizational commitment	5.381	0.791	0.441 **	0.129 **	0.448 **	**0.789**		
Retention intention	4.437	1.465	0.339 **	0.373 **	0.337 **	0.409 **	**0.829**	
Psychological safety	5.543	0.726	0.528 **	0.215 **	0.543 **	0.698 **	0.452 **	**0.784**

Note: values on the diagonal (in bold font) indicate the square root of the AVE for latent variables. ** *p* < 0.05.

**Table 4:** Path coefficients for each variable.

Hypothesis		γ	Result
H1-1	Organizational fit → Organizational commitment	0.649	Supported
H1-2	Organizational links → Organizational commitment	0.607	Supported
H1-3	Organizational sacrifice → Organizational commitment	0.628	Supported
H2	Organizational commitment → Retention intention	0.721	Supported
H3-1	Organizational fit → Retention intention	0.253	Supported
H3-2	Organizational links → Retention intention	0.242	Supported
H3-3	Organizational sacrifice → Retention intention	0.271	Supported

**Table 5:** Mediation effects testing.

Hypothesis		Direct Effect	Indirect Effect	Result
H4-1	Organizational fit→ Retention intention	0.258	0.469	Supported
H4-2	Organizational links→ Retention intention	0.249	0.442	Supported
H4-3	Organizational sacrifice→ Retention intention	0.274	0.455	Supported

**Table 6:** Hierarchical regression analysis findings.

Variables	Retention Intention		
	Model 1	Model 2	Model 3
Step 1: Independent variable			
Organizational commitment	0.717 ***	0.249	0.131
Step 2: Moderator			
Psychological safety		0.625 ***	0.341 ***
Step 3: Retention intention			
Organizational commitment × Psychological safety			0.639 ***
R^2^	0.168	0.242	0.268
ΔR^2^		0.062	0.018
F	45.003 ***	35.371 ***	24.624 ***

Note: *** *p* < 0.001.

## Data Availability

The data that support the findings of this study are available from the corresponding author, H.-S.C., upon reasonable request.
